# The Relationship Between the Average Infusion Rate of Propofol and the Incidence of Delirium During Invasive Mechanical Ventilation: A Retrospective Study Based on the MIMIC IV Database

**DOI:** 10.1111/cns.70273

**Published:** 2025-02-28

**Authors:** Qi‐Yue Ge, Chao Zheng, Xiao‐Bin Song, Zhuang‐Zhuang Cong, Jing Luo, Hao‐Tian Zheng, Peng‐Long Zhao, Yan‐Qing Wang, Bing‐Wei Chen, Yi Shen

**Affiliations:** ^1^ School of Medicine, Southeast University Nanjing China; ^2^ Department of Cardiothoracic Surgery Jinling Hospital Nanjing China; ^3^ Department of Thoracic Surgery National Cancer Center/National Clinical Research Center for Cancer/Cancer Hospital, Chinese Academy of Medical Sciences and Peking Union Medical College Beijing China; ^4^ Jinling Clinical Medical College Nanjing University of Chinese Medicine Nanjing China; ^5^ Department of Cardiothoracic Surgery Jinling Hospital, Affiliated Hospital of Medical School, Nanjing University Nanjing China; ^6^ Department of General Practice Jinling Hospital Nanjing China; ^7^ Department of Biostatistics School of Public Health, Southeast University Nanjing China

**Keywords:** delirium, invasive mechanical ventilation, odds ratio, propofol

## Abstract

**Background:**

Delirium is a common complication observed in intensive care units (ICUs). Propofol is one of the most widely used sedatives and is believed to be closely connected with the incidence of delirium. The study was carried out to explore the relationship between delirium and the average rate of propofol infusion.

**Methods:**

Patients who underwent invasive mechanical ventilation (IMV) while receiving propofol from the Medical Information Mart for Intensive Care IV (MIMIC IV) database were included in the study. The primary outcome was to identify the potential risk factors for the incidence of delirium and investigate the relationship between the average rate of propofol infusion and the incidence of delirium. The secondary outcome was to further analyze the relationship by subgroup analysis. Propensity score matching (PSM) was employed to minimize bias.

**Results:**

A total of 16,956 patients (delirium: 5805; control: 11,151) were ultimately included in the study after PSM. The median diagnostic time of delirium was 18 h. An average propofol infusion rate ≥ 20 μg/(kg*h) during the initial 18 h was found to be independently significant [OR = 1.84, 95% CI = (1.72, 1.98), *p* < 0.001], while an average propofol infusion rate ≤ 40 μg/(kg*h) in the first hour showed no statistically significant difference in the incidence of delirium [OR = 0.95, 95% CI = (0.88, 1.02), *p* = 0.163]. Besides, an average propofol infusion rate ≥ 20 μg/(kg*h) was also found to be statistically significant in all the subgroup analyses.

**Conclusion:**

An average propofol infusion rate ≥ 20 μg/(kg*h) during the initial 18 h was identified as an independent risk factor for delirium, suggesting that the accumulation of propofol might be associated with an increased incidence of delirium.

## Graphical abstract

An average propofol infusion rate ≥ 20 μg/(kg*h) during the initial 18 h of IMV was identified as an independent risk factor for postoperative delirium. This finding was further supported by subgroup analyses.

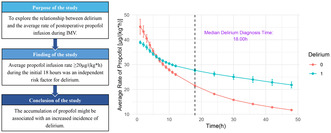

**Abbreviations**
BIDMC: Beth Israel Deaconess Medical Center
CAM‐ICU: Confusion Assessment Method for the Intensive Care Unit
GCS: Glasgow Coma Scale
ICU: intensive care unit
IMV: invasive mechanical ventilation
LODS: logistic organ dysfunction system
MIMIC IV: Medical Information Mart for Intensive Care IV
OASIS: Oxford Acute Severity of Illness Score
OR: odds ratio
PSM: propensity score matching
RCS: restricted cubic splines
SAPS II: Simplified Acute Physiology Score II
SIRS: systemic inflammatory response syndrome
SOFA: sequential organ failure assessment

## 1 Introduction

Delirium, known as an acute confusion state, is a prevalent complication during invasive mechanical ventilation (IMV) [1]. Several factors contribute to the incidence of delirium, including advanced age, exposure to infection, and the use of certain drugs, so that all patients have the risk for delirium. Patients in intensive care units (ICUs), who are typically among the most critically ill in the hospital, might be at an even higher risk for delirium. Sedation is necessary during IMV, and guidelines recommend the use of nonbenzodiazepine sedatives, such as propofol, for critically ill patients undergoing IMV [2]. Propofol, which acts on gamma‐aminobutyric acid receptors, is widely used for sedation and is employed in the induction and maintenance of general anesthesia, as well as in the treatment of delirium due to its notable efficacy [3]. However, propofol was reported to have a potential association with the incidence of delirium [4, 5, 6]. Despite this, the mode by which propofol may induce delirium remains unclear. Therefore, it is important to investigate the relationship between delirium and propofol.

Delirium, characterized by an acute change in mental status, poses significant diagnostic challenges. Many researches have demonstrated that in‐hospital delirium is associated with poor prognosis and reduced postdischarge life quality [7]. Thus, identifying high‐risk patients is crucial for the early detection and management of delirium.

Previous studies have primarily concentrated on comparing propofol with other sedatives, such as midazolam or dexmedetomidine, with limited attention devoted to the correlation between the rate of propofol infusion and the incidence of delirium. The study was launched to identify the high delirium‐risk patients and explore potential strategies to reduce the incidence of delirium.

## 2 Methods and Materials

### 2.1 Study Design

The retrospective study was performed by using data from the Medical Information Mart for Intensive Care IV (MIMIC IV) [8]. MIMIC IV database (version 2.2) contains 76,943 ICU patients between 2008 and 2019 from the Beth Israel Deaconess Medical Center (BIDMC) in Boston, USA.

### 2.2 Patients

ICU patients from BIDMC (via the MIMIC IV database) from 2008 to 2019 were included. The inclusion criteria were listed as follows: (1) ICU patients underwent IMV; (2) patients with records in *procedures_icd* table; and (3) patients with propofol‐using records in hospital. The exclusion criteria were as follows: (1) diagnostic time of delirium before the date of the earliest procedure; (2) the earliest IMV‐starting time before the diagnostic time of delirium; and (3) diagnostic time of delirium before the earliest propofol‐using time.

### 2.3 Data Collection

Delirium patients were diagnosed by CAM‐ICU score. Patients who satisfied feature 1 + 2 + 3 or 1 + 2 + 4 according to the CAM‐ICU scoring system were classified as delirium patients. Patients with IMV‐using were identified by the records of ventilation status. Propofol was distinguished by item ID in “222168.” We defined the average rate of propofol infusion as (total dosage of propofol infusion during the duration/length of the duration).

Data collected from the MIMIC IV database included baseline characteristics included were list as follows: age, gender (male and female), ethnicity (white, black, and other), last care unit (TSICU, MICU/SICU, NICU, CVICU, and CCU), first‐day SOFA, first‐day GCS, SIRS, LODS, OASIS, and SAPS II. The relationship between average rate of propofol infusion and delirium was the primary outcome of the study. Secondary outcomes were in‐hospital mortality and the difference between the high‐delirium risk group and the low‐delirium risk group.

### 2.4 Ethics

We have completed the National Institutes of Health's web‐based course and passed the Protecting Human Research Participants exam (ID: 52932612) to access the MIMIC IV database.

### 2.5 Statistical Analyses

Selected data from the MIMIC IV database was exported as a .xlsx file and then transferred into a .dta file. Stata/SE 16.0 software (Stata Corp, College Station, TX, USA) and R 4.3.3 software (The R Foundation for Statistical Computing, Vienna, Austria; https://www.r‐project.org) were employed to analyze the data. Categorical variables were analyzed by Chi‐squared test and Fisher's exact test. Normally distributed continuous variables were analyzed by *t*‐test and the non‐normal distributed variables were compared by Kruskal–Wallis *H* test. Restricted cubic splines (RCS) were carried out to delineate the potential threshold for a high‐risk amount of propofol. Multiple logistic regression was applied to further analyze the association between characteristics and high‐risk amounts of propofol. A small amount of missing data existed and was replaced by the median. PSM was performed to reduce the bias and the standardized difference figure was used to evaluate the effect of PSM. *p*‐value < 0.05 was considered to be significantly different.

## 3 Results

### 3.1 Baseline Characteristics

**FIGURE 1:**
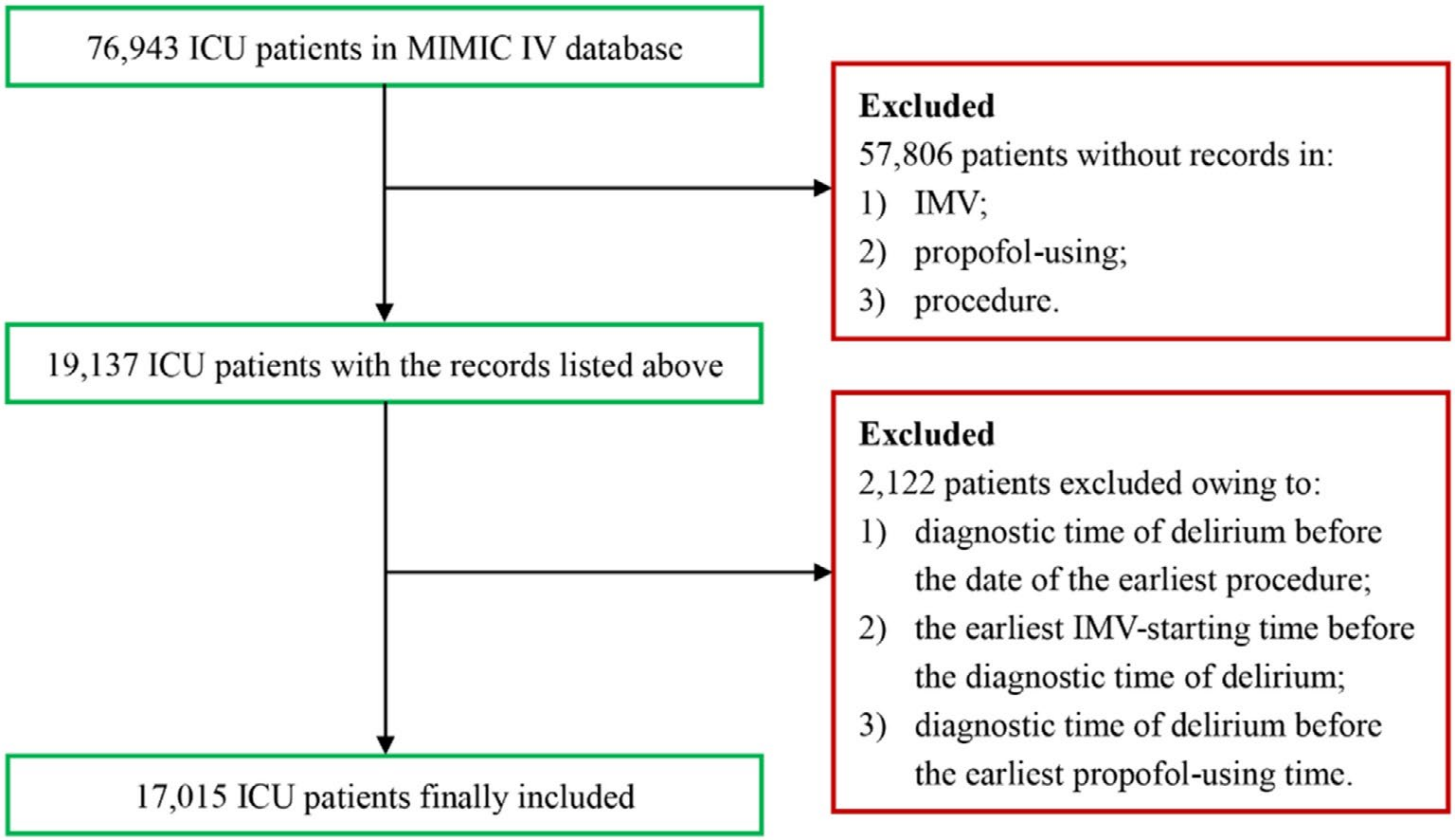
Flowchart of patient selection.

The flowchart of the inclusion is depicted in Figure 1. A comprehensive sample of 17,015 patients (delirium: *n* = 5811; control: *n* = 11,204) was enrolled. Baseline characteristics, including gender, ethnicity, last care unit, first‐day SOFA, first‐day GCS, SIRS, LODS, OASIS, and SAPS II, and clinical information are summarized in Table 1. To minimize the potential bias of the study, PSM was employed to adjust for disparities in baseline characteristics. A refined sample of 16,956 patients was ultimately included after PSM (delirium: *n* = 5805; control: *n* = 11,151). Group demographics and cohort characteristics are listed in Table S1. The assessment of PSM performance is presented in Figure S1.

**TABLE 1:** Group demographics and characteristics.

	Delirium (*n* = 5811)	Control (*n* = 11,204)	*p*
Age, year			
Median, IQR	66.32 (54.89, 75.99)	65.91 (55.29, 75.61)	0.442
Gender			
Female	2380	4165	< 0.001
Male	3431	7039	
Ethnicity			
White	3533	7664	< 0.001
Black	584	796	
Other	1694	2744	
Last care unit			
TSICU	912	1548	< 0.001
MICU/SICU	2899	3923	
NICU	459	147	
CVICU	1151	5137	
CCU	390	449	
First‐day SOFA			
Median, IQR	7 (5, 11)	5 (3, 8)	< 0.001
First‐day GCS			
Median, IQR	12 (8, 14)	14 (10, 15)	< 0.001
SIRS			
Median, IQR	3 (2, 3)	3 (2, 3)	0.807
LODS			
Median, IQR	7 (5, 9)	5 (3, 8)	< 0.001
OASIS			
Median, IQR	39 (34, 45)	36 (31, 41)	< 0.001
SAPS II			
Median, IQR	41 (32, 51)	37 (31, 47)	< 0.001
Average propofol infusion rate, μg/(kg*h)			
1 h			
Median, IQR	37.65 (22.71, 48.92)	40.76 (29.35, 51.78)	< 0.001
2 h			
Median, IQR	36.50 (21.41, 48.62)	40.16 (29.09, 50.98)	< 0.001
3 h			
Median, IQR	34.81 (20.70, 47.80)	39.04 (25.55, 50.04)	< 0.001
4 h			
Median, IQR	32.94 (20.24, 46.52)	35.53 (22.69, 48.09)	< 0.001
5 h			
Median, IQR	31.57 (19.98, 45.24)	32.38 (20.30, 45.59)	0.017
6 h			
Median, IQR	30.49 (19.35, 43.93)	30.08 (18.79, 43.24)	0.220
7 h			
Median, IQR	29.96 (18.44, 42.85)	28.30 (17.01, 41.48)	< 0.001
8 h			
Median, IQR	29.38 (17.51, 42.22)	26.32 (15.45, 40.33)	< 0.001
9 h			
Median, IQR	28.69 (16.89, 41.47)	24.75 (14.14, 39.46)	< 0.001
10 h			
Median, IQR	28.07 (16.15, 40.78)	23.25 (13.02. 38.28)	< 0.001
11 h			
Median, IQR	27.41 (15.45, 40.32)	21.88 (12.00, 37.11)	< 0.001
12 h			
Median, IQR	26.92 (14.91, 39.99)	20.79 (11.13, 36.06)	< 0.001
18 h			
Median, IQR	23.74 (11.99, 36.75)	15.71 (7.79, 30.08)	< 0.001
24 h			
Median, IQR	21.08 (10.11, 33.76)	12.32 (5.90, 25.33)	< 0.001
30 h			
Median, IQR	19.08 (8.52, 31.78)	10.12 (4.77, 21.53)	< 0.001
36 h			
Median, IQR	17.17 (7.42, 29.78)	8.53 (4.00, 18.82)	< 0.001
42 h			
Median, IQR	15.55 (6.52, 27.81)	7.38 (3.44, 16.63)	< 0.001
48 h			
Median, IQR	14.05 (5.83, 26.33)	6.50 (3.02, 14.77)	< 0.001
In‐hospital mortality			
Yes	953	1452	< 0.001
No	4858	9752	

### 3.2 Primary Outcomes

**FIGURE 2:**
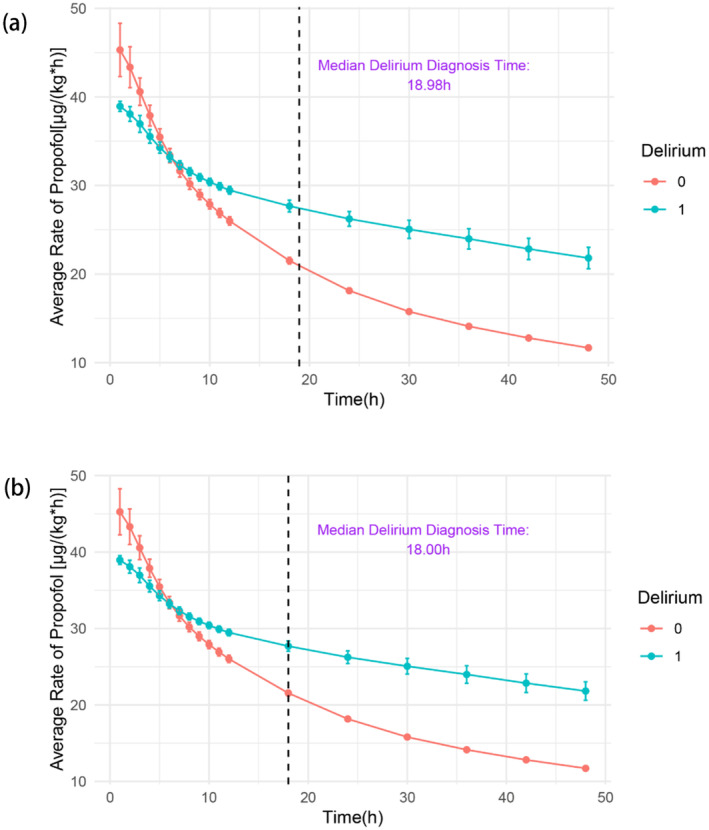
Visualization of the average rate of propofol infusion. (a) Average rate of propofol infusion before PSM. Median delirium diagnosis time after the first postoperative propofol infusion was 18.89 h; (b) Average rate of propofol infusion after PSM. Median delirium diagnosis time after the first postoperative propofol infusion was 18.00 h.

The disparities in the average propofol infusion rate between the delirium and control groups are graphically presented in Figure 2. Patients belonging to the delirium group exhibited a notably higher average propofol infusion rate in the initial 5 h of IMV, followed by a decrease to a lower rate subsequent to 7 h. Specifically, the median time to diagnose delirium was the 18th hour.

**FIGURE 3:**
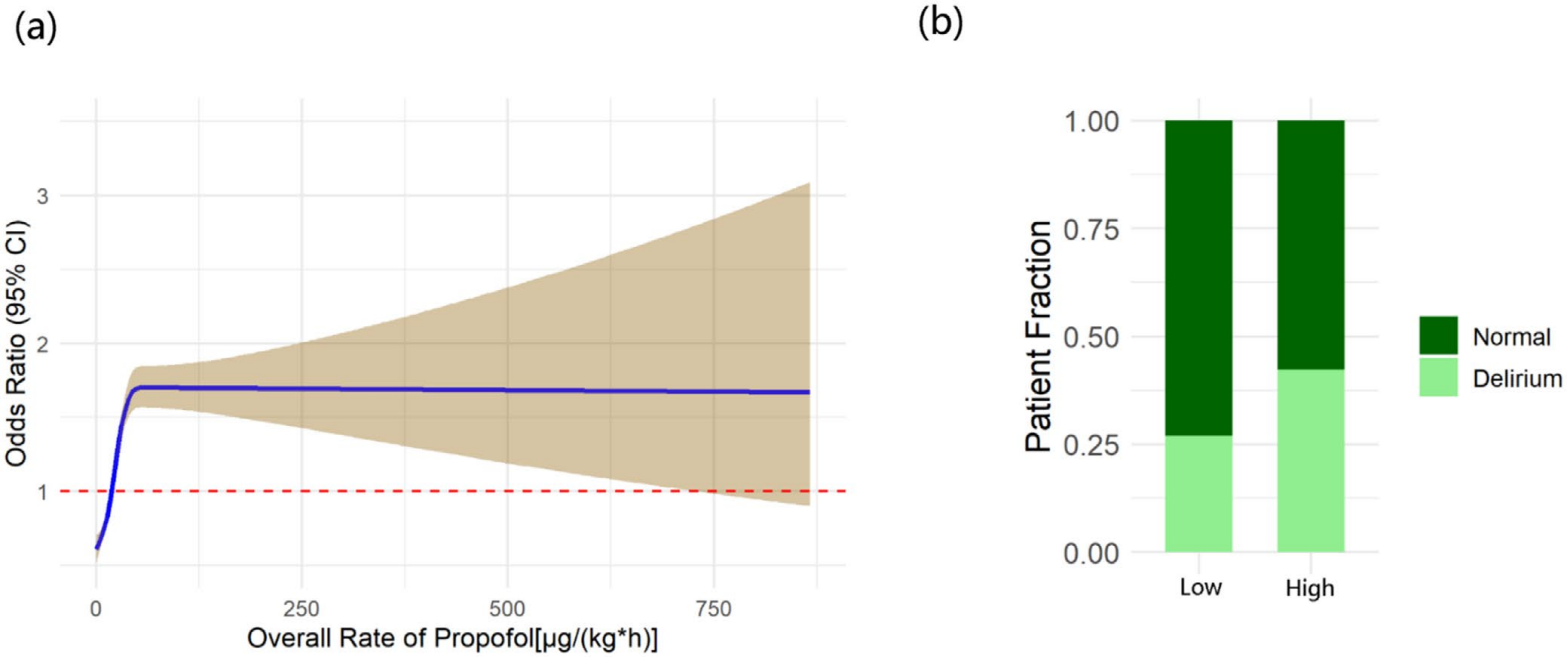
RCS plot and stacked bar chart of the initial 18 h. (a) RCS plot of the initial 18 h. Average propofol infusion rate of ≥ 20 μg/(kg*h) in the initial 18 h was selected as an indicator to differentiate between high and low risks of delirium; (b) Stacked bar chart showing the proportion of delirium patients in high‐risk and low‐risk groups during the first 18 h.

Following the application of RCS, 40 μg/(kg*h) within the first hour and 20 μg/(kg*h) within the initial 18 h were selected as the indicators to differentiate between high and low risks of delirium, respectively (Figure S2a and Figure 3). Additionally, stacked bar charts depicting these data are also shown in the figures (Figure S2b and Figure 3b). A higher proportion of delirium patients was observed in the group that received a higher average rate of propofol infusion.

In the multiple logistic regression model, a higher incidence of delirium was observed to be associated with an average propofol infusion rate exceeding 20 μg/(kg*h) during the initial 18 h. However, an average propofol infusion rate below 40 μg/(kg*h) within the first hour did not demonstrate any statistically significant difference in the incidence of delirium. Multicollinearity was also rigorously assessed, and the variables were appropriately adjusted accordingly (Tables S2 and S3). Notably, the results remained consistent in both 18 h model [OR = 1.84, 95% CI = (1.72, 1.98), *p* < 0.001] and 1 h model [OR = 0.95, 95% CI = (0.88, 1.02), *p* = 0.163] after adjustment (Table 2 and Table S4).

**TABLE 2:** Multiple logistic regression of delirium (18 h).

Delirium	OR	Standard error	*z*	*p* > |*z*|	95% CI
Age, years					
(< 60)					
≥ 60	1.318194	0.0506589	7.19	< 0.001	[1.222551, 1.421318]
Gender					
(Female)					
Male	0.9047045	0.0327836	−2.76	0.006	[0.8426785, 0.9712958]
Race					
(Other)					
White	0.8371806	0.0339406	−4.38	< 0.001	[0.7732326, 0.9064172]
Black	1.092213	0.0741012	1.30	0.194	[0.9562191, 1.247547]
Last care unit					
(TSICU)					
MICU/SICU	1.013774	0.0515744	0.27	0.788	[0.9175658, 1.120069]
NICU	6.082144	0.6461928	16.99	< 0.001	[4.9388, 7.490175]
CVICU	0.3574196	0.020043	−18.35	< 0.001	[0.3202179, 0.3989432]
CCU	1.122617	0.095265	1.36	0.173	[0.9506026, 1.325759]
First‐day SOFA	1.126682	0.0051812	25.94	< 0.001	[1.116572, 1.136883]
High‐risk (18 h), μg/(kg*h)					
(< 20)					
≥ 20	1.844288	0.0656746	17.19	< 0.001	[1.719957, 1.977606]
Constant	0.2103473	0.0142313	−23.04	< 0.001	[0.1842247, 0.240174]

### 3.3 Secondary Outcomes

**FIGURE 4:**
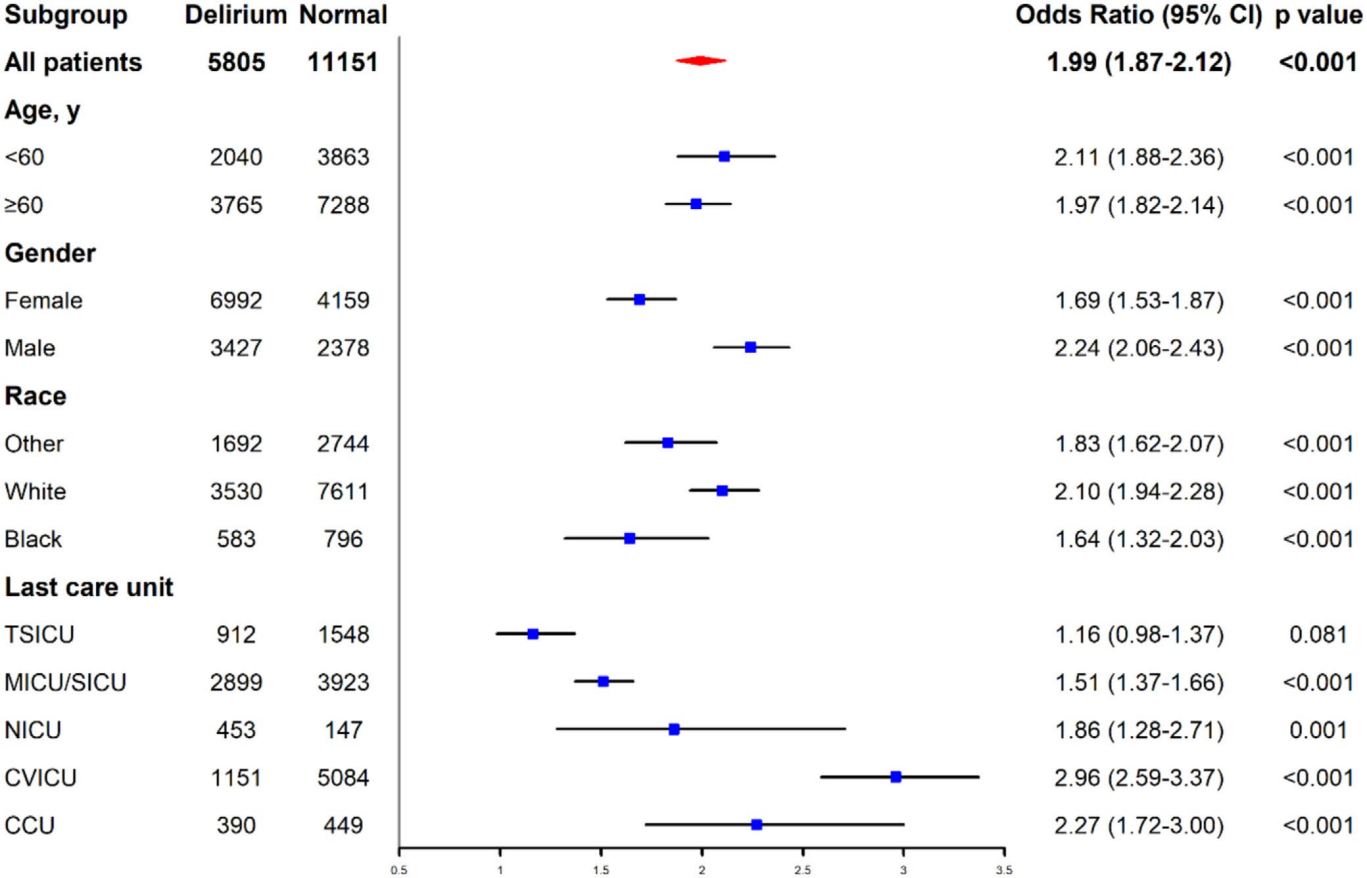
Forest plot of the subgroup analysis. Subgroup analyses for age, gender, race, and last care unit. Logistic regression model was used to assess the impact of average propofol infusion rate ≥ 20 μg/(kg*h) in the initial 18 h.

Subgroup analysis of the logistic regression revealed that average propofol amount exceeding 20 μg/(kg*h) had close connection in specific characteristics of the population, including age (< 60) [OR = 2.11, 95% CI = (1.88, 2.36), *p* < 0.001], age (≥ 60) [OR = 1.97, 95% CI = (1.82, 2.14), *p* < 0.001], gender (female) [OR = 1.69, 95% CI = (1.53, 1.87), *p* < 0.001], gender (male) [OR = 2.24, 95% CI = (2.06, 2.43), *p* < 0.001], race (white) [OR = 2.10, 95% CI = (1.94, 2.28), *p* < 0.001], race (black) [OR = 1.64, 95% CI = (1.32, 2.03), *p* < 0.001], race (other) [OR = 1.83, 95% CI = (1.62, 2.07), *p* < 0.001], last care unit (TSICU) [OR = 1.16, 95% CI = (0.98, 1.37), *p* = 0.081], last care unit (MICU/SICU) [OR = 1.51, 95% CI = (1.37, 1.66), *p* < 0.001], last care unit (NICU) [OR = 1.86, 95% CI = (1.28, 2.71), *p* = 0.001], last care unit (CVICU) [OR = 2.96, 95% CI = (2.59, 3.37), *p* < 0.001], and last care unit (CCU) [OR = 2.27, 95% CI = (1.72, 3.00), *p* < 0.001]. The forest plot is shown in Figure 4.

## 4 Discussion

The results indicated that patients with delirium had a higher average rate of propofol infusion during the first 18 h, suggesting that an average propofol infusion rate of ≥ 20 μg/(kg*h) in the initial 18 h was a significant risk factor for delirium. The hypothesis was supported by the multiple logistic regression model. Conversely, although Figure 2 demonstrates differences both at the first hour and during the initial 18 h, the average propofol infusion rate under 40 μg/(kg*h) in the first hour was found not to be associated with the higher incidence of delirium in another multiple logistic regression model. The findings suggested that the accumulation of propofol, rather than a higher momentary rate, might be the actual contributing factor to the increased incidence of delirium. A similar result was observed in a previous study on anesthesia induction. No significant difference in delirium incidence was found between varying propofol injection rates [9]. Additionally, as depicted in Table 1, the incidence of delirium was connected to a higher in‐hospital mortality (*p* < 0.001), indicating that reducing the accumulation of propofol might potentially decrease the in‐hospital mortality.

The results indicated that, in addition to the average propofol infusion rate, age, gender, race, last care unit, and first‐day SOFA were also associated with the incidence of delirium. Previous studies have generally identified the elderly as a risk factor for delirium [10, 11]. The SOFA score was designed to evaluate the degree of organ dysfunction or failure and the current research depicts that the SOFA score is a risk factor for both delirium and in‐hospital mortality [12]. Besides, gender was also recognized as an important factor in the incidence of delirium in prior studies. An investigation in the relationship between delirium and gender found that delirium would more frequently affect female patients over 85 years old in the cardiac ICU, which might explain the difference in gender observed in the multiple logistic regression model [13]. Additionally, patients in the NICU exhibited a higher incidence of delirium. One potential explanation was that the neurological disease itself could result in delirium by inducing neurological inflammation and other mechanisms, making NICU residency a significant factor in the incidence of delirium [14, 15]. In contrast to the findings in cardiovascular surgery [16, 17, 18], although patients in CVICU showed similar outcomes as other ICUs during subgroup analysis, CVICU appeared to have a lower impact on delirium compared to other ICUs. This phenomenon might be attributed to the low proportion of patients who underwent extracorporeal circulation and open‐heart operation.

To deal with the high incidence of delirium associated with propofol, several researches paid attention to the alternatives. Dexmedetomidine has also been widely utilized in patients undergoing IMV. Evidence‐based studies revealed that dexmedetomidine might shorten the duration of IMV and reduce the incidence of delirium [4, 5]. However, dexmedetomidine might also increase the risk of cardiac events [19]. Another commonly used sedative was midazolam. Previous researches depicted that although midazolam exhibited similar sedative efficacy, it was associated with higher mortality and an increased incidence of delirium [20, 21]. Future studies should focus on alternative sedatives with both a lower incidence of delirium and safety.

In the treatment of delirium, pharmacological intervention was generally considered as a common practice in delirium treatment. However, recent researches demonstrated that drugs like haloperidol and other antipsychotics might not be as effective as expected. Prevention was considered to be the most optimal treatment. Nonpharmacologic interventions have been important approaches in the past few decades and were recommended by clinical guidelines [14, 22, 23]. Future treatment for delirium should concentrate on the combination of pharmacologic therapies, nonpharmacologic prevention, and monitoring.

A key strength of our study was that it was the first study to concentrate on the relationship between average propofol infusion rate and the incidence of delirium in patients undergoing IMV. Furthermore, the research included a relatively large sample size. The results of this study suggested that the role of the average propofol infusion rate during the initial 18 h made great sense to the incidence of delirium, which might contribute to the future management of ICU patients undergoing IMV.

Some limitations also existed in the study. Initially, the average rate of propofol infusion was calculated based on the weight, time, and total amount of propofol, which made it difficult to explore the relationship between delirium and temporary propofol infusion rate. Secondarily, the effects of other drugs were not comprehensively considered, though the influence of propofol was demonstrated by multiple logistic regression. Additionally, it was difficult to identify different subtypes of delirium within the MIMIC IV database, which should be taken into consideration in future studies.

## 5 Conclusion

An average propofol infusion rate ≥ 20 μg/(kg*h) during the initial 18 h was an independent risk factor for delirium, suggesting that the accumulation of propofol might be associated with an increased incidence of delirium. Future studies should focus on the different subtypes of delirium and comprehensive management of delirium.

## Author Contributions

Qi‐Yue Ge, Chao Zheng, and Yi Shen completed the conceptualization. The methodology was finished by Qi‐Yue Ge, Chao Zheng, Zhuang‐Zhuang Cong, Yan‐Qing Wang, Bing‐Wei Chen, and Yi Shen. The formal analysis was accomplished by Qi‐Yue Ge and Chao Zheng. The investigation was contributed by Qi‐Yue Ge, Chao Zheng, Jing Luo, Xiao‐Bin Song, Hao‐Tian Zheng, and Peng‐Long Zhao. The data curation was done by Qi‐Yue Ge and Chao Zheng. The visualization was achieved by Qi‐Yue Ge and Chao Zheng. Zhuang‐Zhuang Cong, Jing Luo, and Yi Shen made great contributions to the funding acquisition. Resources were completed by Qi‐Yue Ge, Chao Zheng, and Yi Shen. Supervision was given by Yan‐Qing Wang, Bing‐Wei Chen, and Yi Shen. All authors wrote and/or reviewed the manuscript.

## Ethics Statement

We have completed the National Institutes of Health's web‐based course and passed the Protecting Human Research Participants exam (ID: 52932612) to access the MIMIC IV database; Due to the retrospective nature, the study was dispensed from obtaining individual inform consents.

## Consent

The authors have nothing to report.

## Conflicts of Interest

The authors declare no conflicts of interest.

## Supporting information


Figure S1.

Figure S2.

Table S1.

Table S2.

Table S3.

Table S4.

## Data Availability

Dataset of the study could be obtained from the MIMIC IV database (https://mimic.mit.edu).
