# Supplementary figures and images for: The Relationship Between the Average Infusion Rate of Propofol and the Incidence of Delirium During Invasive Mechanical Ventilation: A Retrospective Study Based on the MIMIC IV Database
Source: CNS Neurosci Ther. 2025 Feb 28;31(3):e70273. doi: 10.1111/cns.70273 (PMC11868985; doi:10.1111/cns.70273)

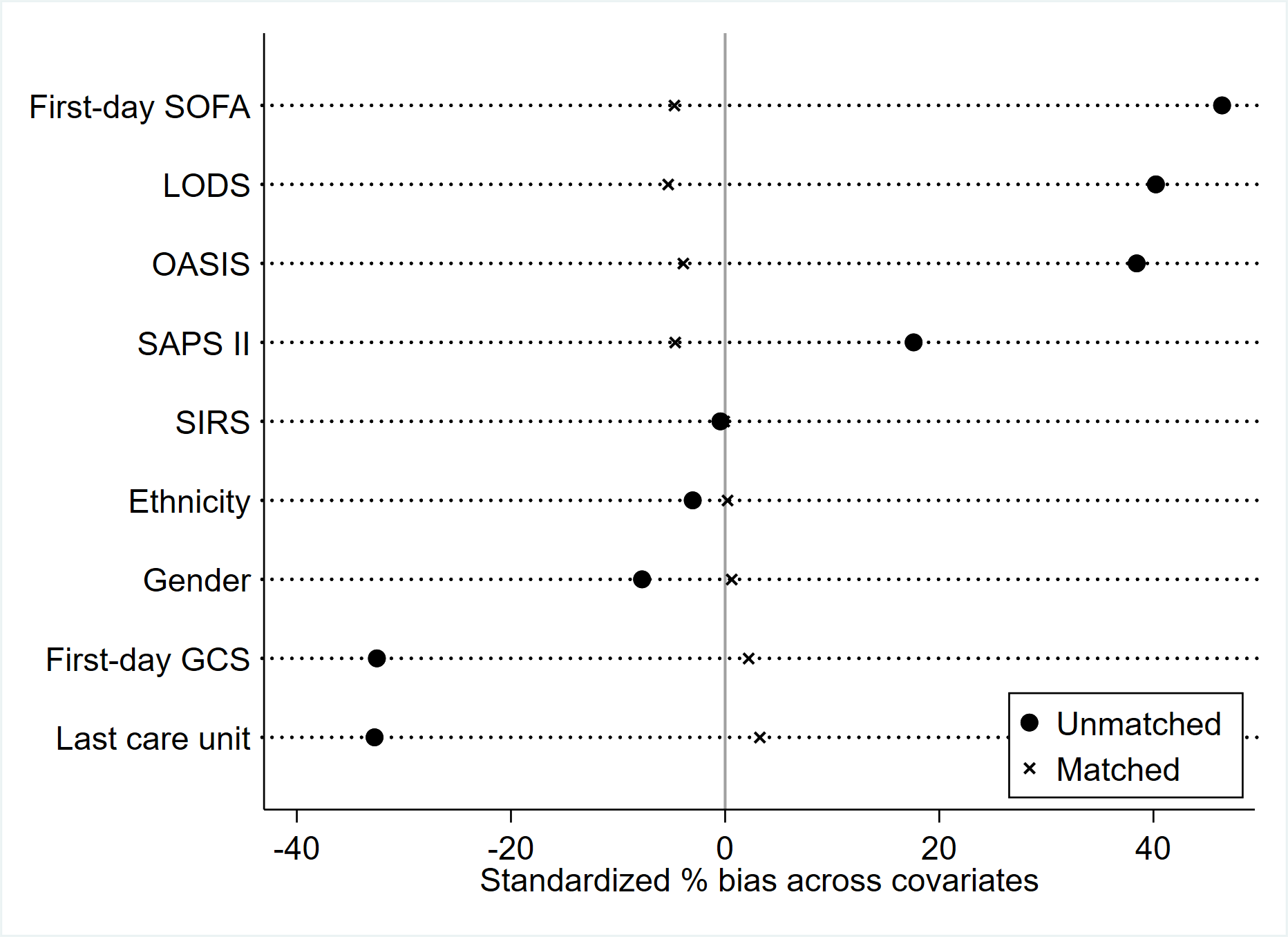

Supplement: Supplementary file 1 — Figure S1. [file CNS-31-e70273-s006.tif]

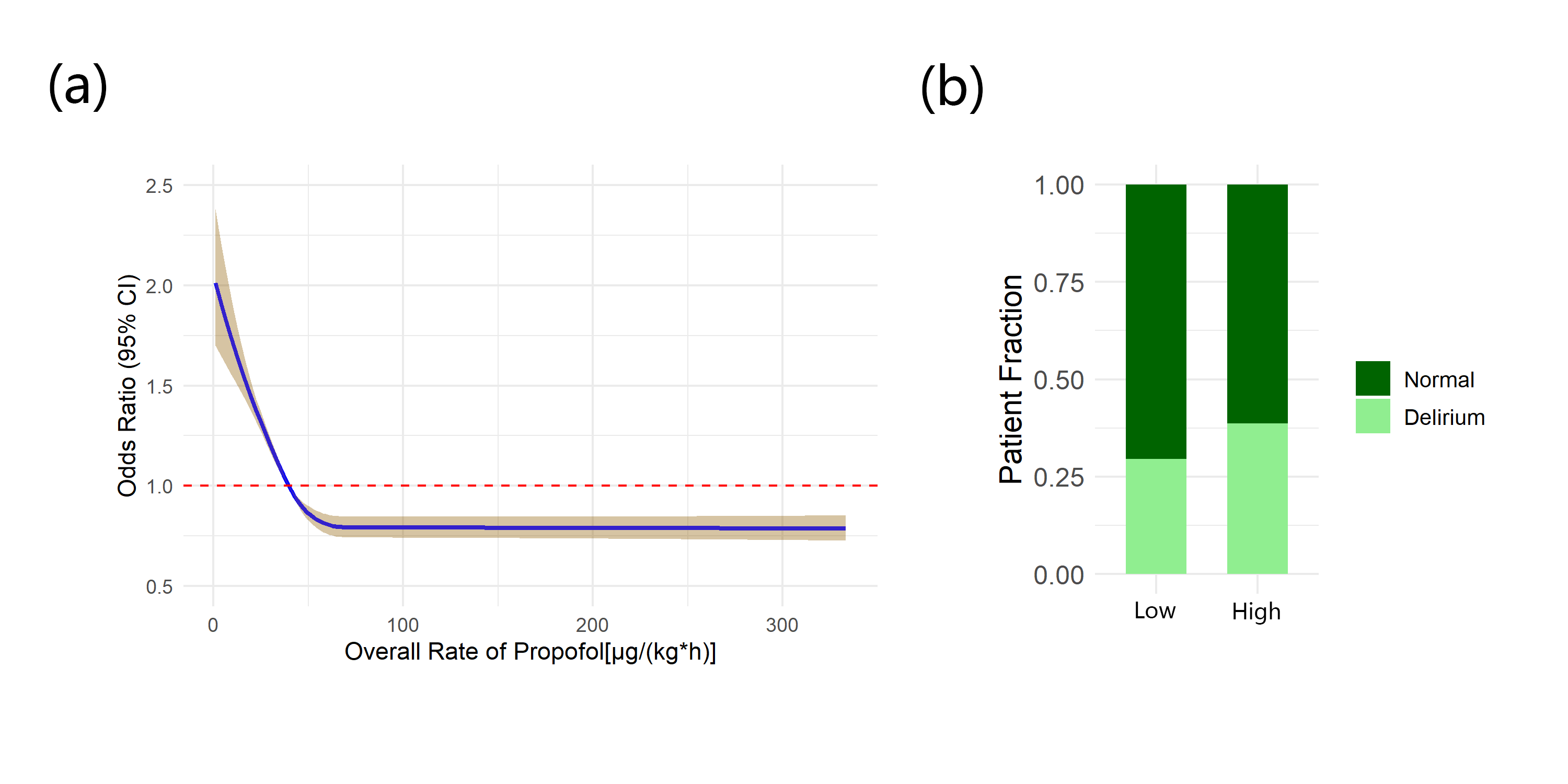

Supplement: Supplementary file 2 — Figure S2. [file CNS-31-e70273-s004.tif]
