# Supplementary material for: The Relationship Between the Average Infusion Rate of Propofol and the Incidence of Delirium During Invasive Mechanical Ventilation: A Retrospective Study Based on the MIMIC IV Database
Source: CNS Neurosci Ther. 2025 Feb 28;31(3):e70273. doi: 10.1111/cns.70273 (PMC11868985; doi:10.1111/cns.70273)
Supplement: Supplementary file 3 — Table S1. [file CNS-31-e70273-s005.docx]

**Supplementary Table 1.** Group demographics and cohort characteristics (after PSM)

|  | Delirium | Control |
| --- | --- | --- |
|  | (n=5,805) | (n=11,151) |
| Age, year |  |  |
| Median, IQR | 65.90 (55.28, 75.62) | 66.34 (54.91, 75.99) |
| Gender |  |  |
| Female | 2,378 | 4,159 |
| Male | 3,427 | 6,992 |
| Ethnicity |  |  |
| White | 3,530 | 7,611 |
| Black | 583 | 796 |
| Other | 1,692 | 2,744 |
| Last Care Unit |  |  |
| TSICU | 912 | 1,548 |
| MICU/SICU | 2,899 | 3,923 |
| NICU | 453 | 147 |
| CVICU | 1,151 | 5,084 |
| CCU | 390 | 449 |
| First-day SOFA |  |  |
| Median, IQR | 7 (5, 11) | 5 (4, 8) |
| First-day GCS |  |  |
| Median, IQR | 12 (8, 14) | 14 (10, 15) |
| SIRS |  |  |
| Median, IQR | 3 (2, 3) | 3 (2, 3) |
| LODS |  |  |
| Median, IQR | 7 (5, 9) | 5 (3, 8) |
| OASIS |  |  |
| Median, IQR | 39 (34, 45) | 36 (31, 41) |
| SAPS II |  |  |
| Median, IQR | 41 (32, 51) | 37 (31, 47) |
| Average propofol infusion rate, μg/(kg*h) |  |  |
| 1h |  |  |
| Median, IQR | 37.66 (22.71, 48.92) | 40.72 (29.30, 51.74) |
| 2h |  |  |
| Median, IQR | 36.52 (21.43, 48.63) | 40.14 (29.06, 50.94) |
| 3h |  |  |
| Median, IQR | 34.81 (20.70, 47.80) | 38.99 (25.49, 50.03) |
| 4h |  |  |
| Median, IQR | 32.97 (20.25, 46.52) | 35.49 (22.64, 48.08) |
| 5h |  |  |
| Median, IQR | 31.58 (20.00, 45.24) | 32.38 (20.27, 45.59) |
| 6h |  |  |
| Median, IQR | 30.49 (19.35, 43.93) | 30.10 (18.76, 43.28) |
| 7h |  |  |
| Median, IQR | 29.96 (18.44, 42.85) | 28.31 (16.98, 41.50) |
| 8h |  |  |
| Median, IQR | 29.41 (17.51, 42.22) | 26.36 (15.43, 40.34) |
| 9h |  |  |
| Median, IQR | 28.70 (16.89, 41.47) | 24.77 (14.13, 39.48) |
| 10h |  |  |
| Median, IQR | 28.08 (16.15, 40.78) | 23.31 (13.01. 38.33) |
| 11h |  |  |
| Median, IQR | 27.41 (15.46, 40.32) | 21.92 (12.00, 37.16) |
| 12h |  |  |
| Median, IQR | 26.93 (14.95, 39.99) | 20.82 (11.13, 36.12) |
| 18h |  |  |
| Median, IQR | 23.77 (12.02, 36.75) | 15.77 (7.79, 30.12) |
| 24h |  |  |
| Median, IQR | 21.09 (10.12, 33.77) | 12.38 (5.90, 25.38) |
| 30h |  |  |
| Median, IQR | 19.09 (8.52, 31.78) | 10.16 (4.77, 21.61) |
| 36h |  |  |
| Median, IQR | 17.18 (7.43, 29.78) | 8.57 (4.00, 18.88) |
| 42h |  |  |
| Median, IQR | 15.55 (6.52, 27.81) | 7.42 (3.44, 16.68) |
| 48h |  |  |
| Median, IQR | 14.05 (5.83, 26.33) | 6.52 (3.02, 14.83) |
| In-hospital Mortality |  |  |
| Yes | 952 | 1,451 |
| No | 4,853 | 9,700 |
