# Supplementary material for: The Relationship Between the Average Infusion Rate of Propofol and the Incidence of Delirium During Invasive Mechanical Ventilation: A Retrospective Study Based on the MIMIC IV Database
Source: CNS Neurosci Ther. 2025 Feb 28;31(3):e70273. doi: 10.1111/cns.70273 (PMC11868985; doi:10.1111/cns.70273)
Supplement: Supplementary file 4 — Table S2. [file CNS-31-e70273-s002.docx]

**Supplementary table 2.** multicollinearity test of the multiple logistic regression (18h)

| Variable | VIF | 1/VIF |
| --- | --- | --- |
| Age | 3.95 | 0.253453 |
| Gender | 2.6 | 0.384609 |
| Race |  |  |
| White | 3.52 | 0.284096 |
| Black | 1.31 | 0.761136 |
| Last care unit |  |  |
| MICU/SICU | 3.7 | 0.270333 |
| NICU | 1.22 | 0.820159 |
| CVICU | 3.54 | 0.282191 |
| CCU | 1.37 | 0.732225 |
| First-day GCS | 8.91 | 0.112219 |
| SIRS | 11.48 | 0.087117 |
| First-day SOFA | 10.57 | 0.094635 |
| SAPS II | 25.39 | 0.03939 |
| OASIS | 32.43 | 0.03084 |
| LODS | 17.59 | 0.056862 |
| High-risk (18h) | 1.93 | 0.518129 |
| Mean VIF | 8.63 |  |
