# Supplementary material for: The Relationship Between the Average Infusion Rate of Propofol and the Incidence of Delirium During Invasive Mechanical Ventilation: A Retrospective Study Based on the MIMIC IV Database
Source: CNS Neurosci Ther. 2025 Feb 28;31(3):e70273. doi: 10.1111/cns.70273 (PMC11868985; doi:10.1111/cns.70273)
Supplement: Supplementary file 5 — Table S3. [file CNS-31-e70273-s001.docx]

**Supplementary Table 3.** Multicollinearity of multiple logistic regression (1h)

| Variable | VIF | 1/VIF |
| --- | --- | --- |
| Age | 3.97 | 0.251597 |
| Gender | 2.60 | 0.385204 |
| Race |  |  |
| White | 3.51 | 0.284530 |
| Black | 1.31 | 0.761847 |
| Last care unit |  |  |
| MICU/SICU | 3.70 | 0.270427 |
| NICU | 1.22 | 0.819902 |
| CVICU | 3.61 | 0.276626 |
| CCU | 1.37 | 0.731916 |
| First-day GCS | 8.82 | 0.113442 |
| SIRS | 11.48 | 0.087138 |
| First-day SOFA | 10.54 | 0.094911 |
| SAPS II | 25.29 | 0.039537 |
| OASIS | 32.48 | 0.030791 |
| LODS | 17.59 | 0.056836 |
| High-risk (1h) | 2.32 | 0.431429 |
| Mean VIF | 8.65 |  |
