# Supplementary material for: The Relationship Between the Average Infusion Rate of Propofol and the Incidence of Delirium During Invasive Mechanical Ventilation: A Retrospective Study Based on the MIMIC IV Database
Source: CNS Neurosci Ther. 2025 Feb 28;31(3):e70273. doi: 10.1111/cns.70273 (PMC11868985; doi:10.1111/cns.70273)
Supplement: Supplementary file 6 — Table S4. [file CNS-31-e70273-s003.docx]

**Supplementary Table 4.** Multiple logistic regression of delirium (1h)

| Delirium | OR | Standard Error | z | P> \|z\| | 95% CI |
| --- | --- | --- | --- | --- | --- |
| Age, y |  |  |  |  |  |
| (<60) |  |  |  |  |  |
| ≥60 | 1.213077 | 0.0463052 | 5.06 | <0.001 | [1.125633, 1.307315] |
| Gender |  |  |  |  |  |
| (Female) |  |  |  |  |  |
| Male | 0.9228723 | 0.0332058 | -2.23 | 0.026 | [0.860032, 0.9903043] |
| Race |  |  |  |  |  |
| (Other) |  |  |  |  |  |
| White | 0.8434865 | 0.0339114 | -4.23 | <0.001 | [0.7795726, 0.9126404] |
| Black | 1.102368 | 0.0741944 | 1.45 | 0.148 | [0.9661326, 1.257814] |
| Last care unit |  |  |  |  |  |
| (TSICU) |  |  |  |  |  |
| MICU/SICU | 0.994425 | 0.0503064 | -0.11 | 0.912 | [0.9005567, 1.098077] |
| NICU | 5.955647 | 0.6281658 | 16.92 | <0.001 | [4.843389, 7.32333] |
| CVICU | 0.334327 | 0.0188337 | -19.45 | <0.001 | [0.2993784, 0.3733554] |
| CCU | 1.087097 | 0.0914213 | 0.99 | 0.321 | [0.9219029, 1.281893] |
| First-day SOFA | 1.129343 | 0.0052475 | 26.18 | <0.001 | [1.119104, 1.139674] |
| High-risk (1h), μg/(kg*h) |  |  |  |  |  |
| (<40) |  |  |  |  |  |
| ≥40 | 0.9498444 | 0.0349946 | -1.40 | 0.163 | [0.8836741, 1.02097] |
| Constant | 0.311337 | 0.0202061 | -17.98 | <0.001 | [0.2741492, 0.3535693] |
